# Supplementary material for: COZOID: contact zone identifier for visual analysis of protein-protein interactions
Source: BMC Bioinformatics. 2018 Apr 6;19:125. doi: 10.1186/s12859-018-2113-6 (PMC5889581; doi:10.1186/s12859-018-2113-6)
Supplement: Supplementary file 2 — Software build. Executable binary file of the software tool. (ZIP 118,784 kb) [file 12859_2018_2113_MOESM2_ESM.zip › cozoid/java/docs/beansbinding-1.2.1-doc.zip!/allclasses-frame.html]

All Classes (Beans Binding)


**All Classes**
  

AbstractBindingListener
  
AbstractButtonAdapterProvider
  
AbstractButtonAdapterProvider.Adapter
  
AbstractColumnBinding
  
ArithmeticNode
  
ArrayELResolver
  
AstAnd
  
AstBracketSuffix
  
AstChoice
  
AstCompositeExpression
  
AstDeferredExpression
  
AstDiv
  
AstDotSuffix
  
AstDynamicExpression
  
AstEmpty
  
AstEqual
  
AstFalse
  
AstFloatingPoint
  
AstFunction
  
AstGreaterThan
  
AstGreaterThanEqual
  
AstIdentifier
  
AstInteger
  
AstLessThan
  
AstLessThanEqual
  
AstLiteralExpression
  
AstMinus
  
AstMod
  
AstMult
  
AstNegative
  
AstNot
  
AstNotEqual
  
AstNull
  
AstOr
  
AstPlus
  
AstString
  
AstTrue
  
AstValue
  
AstValue.Target
  
AutoBinding
  
AutoBinding.UpdateStrategy
  
BeanAdapterBase
  
BeanAdapterFactory
  
*BeanAdapterProvider*
  
BeanELResolver
  
BeanELResolver.BeanProperties
  
BeanELResolver.BeanProperty
  
BeanProperty
  
Binding
  
Binding.SyncFailure
  
Binding.SyncFailureType
  
Binding.ValueResult
  
BindingGroup
  
*BindingListener*
  
Bindings
  
BooleanNode
  
CompositeELResolver
  
Converter
  
ELArithmetic
  
ELArithmetic.BigDecimalDelegate
  
ELArithmetic.BigIntegerDelegate
  
ELArithmetic.DoubleDelegate
  
ELArithmetic.LongDelegate
  
ELContext
  
ELContextEvent
  
*ELContextListener*
  
ELException
  
ELParser
  
*ELParserConstants*
  
ELParserTokenManager
  
*ELParserTreeConstants*
  
ELProperty
  
ELResolver
  
ELSupport
  
EvaluationContext
  
Expression
  
Expression.ResolvedProperty
  
Expression.Result
  
Expression.Result.Type
  
ExpressionBuilder
  
ExpressionFactory
  
ExpressionFactoryImpl
  
FunctionMapper
  
FunctionMapperFactory
  
FunctionMapperImpl
  
FunctionMapperImpl.Function
  
JComboBoxAdapterProvider
  
JComboBoxAdapterProvider.Adapter
  
JComboBoxBinding
  
JListAdapterProvider
  
JListBinding
  
JSliderAdapterProvider
  
JSliderAdapterProvider.Adapter
  
JSpinnerAdapterProvider
  
JSpinnerAdapterProvider.Adapter
  
JTableAdapterProvider
  
JTableBinding
  
JTextComponentAdapterProvider
  
ListBindingManager
  
ListELResolver
  
MapELResolver
  
MessageFactory
  
MethodExpression
  
MethodExpressionImpl
  
MethodExpressionLiteral
  
MethodInfo
  
MethodNotFoundException
  
*Node*
  
*NodeVisitor*
  
ObjectProperty
  
ObservableCollections
  
ObservableCollections.ObservableListHelper
  
*ObservableList*
  
*ObservableListListener*
  
*ObservableMap*
  
*ObservableMapListener*
  
ParseException
  
Property
  
PropertyHelper
  
PropertyNotFoundException
  
PropertyNotWritableException
  
PropertyResolutionException
  
PropertyStateEvent
  
*PropertyStateListener*
  
ReflectionUtil
  
ResourceBundleELResolver
  
SimpleCharStream
  
SimpleNode
  
SwingBindings
  
Token
  
TokenMgrError
  
Validator
  
ValueExpression
  
ValueExpressionImpl
  
ValueExpressionLiteral
  
VariableMapper
  
VariableMapperFactory
  
VariableMapperImpl
